# Gpx3 and Egr1 Are Involved in Regulating the Differentiation Fate of Cardiac Fibroblasts under Pressure Overload

**DOI:** 10.1155/2022/3235250

**Published:** 2022-06-28

**Authors:** Guoxing Li, Yuhong Qin, Zhe Cheng, Xiaocheng Cheng, Ruiyu Wang, Xuexiu Luo, Yipin Zhao, Dongying Zhang, Gang Li

**Affiliations:** ^1^Department of Cardiology, The First Affiliated Hospital of Chongqing Medical University, Chongqing 400016, China; ^2^Department of Hepatology and Translational Medicine, Chongqing University Fuling Hospital, Chongqing 400016, China; ^3^Department of Cardiology, Chongqing University Three Gorges Hospital, Chongqing 404199, China; ^4^Department of Critical Medicine, Fuqing Hospital Affiliated to Fujian Medical University, Fuqing 350300, China; ^5^Department of Cardiology, The First Affiliated Hospital of USTC, Division of Life Sciences and Medicine, University of Science and Technology of China, Hefei 230001, China; ^6^Institute of Life Sciences, Chongqing Medical University, 400016, China; ^7^Molecular Medicine Diagnostic and Testing Center, Chongqing Medical University, 400016, China

## Abstract

**Objectives:**

Although myocardial fibrosis is a common pathophysiological process associated with many heart diseases, the molecular mechanisms regulating the development of fibrosis have not been fully determined. Recently, single cell RNA sequencing (scRNA-seq) analysis has been used to examine cellular fate and function during cellular differentiation and has contributed to elucidating the mechanisms of various diseases. The main purpose of this study was to characterize the fate of cardiac fibroblasts (CFs) and the dynamic gene expression patterns in a model of cardiac pressure overload using scRNA-seq analysis.

**Methods:**

The public scRNA-seq dataset of the transverse aortic coarctation (TAC) model in mice was downloaded from the GEO database, GSE155882. First, we performed quality control, dimensionality reduction, clustering, and annotation of the data through the Seurat R package (v4.0.5). Then, we constructed the pseudotime trajectory of cell development and identified key regulatory genes using the Monocle R package (v2.22.0). Different cell fates and groups were fully characterized by Gene Set Enrichment Analysis (GSEA) analysis and Transcription factor (TF) activity analysis. Finally, we used Cytoscape (3.9.1) to extensively examine the gene regulatory network related to cell fate.

**Results:**

Pseudotime analysis showed that CFs differentiated into two distinct cell fates, one of which produced activated myofibroblasts, and the other which produced protective cells that were associated with reduced fibrosis levels, increased antioxidative stress responses, and the ability to promote angiogenesis. In the TAC model, activated CFs were significantly upregulated, while protective cells were downregulated. Treatment with the bromodomain inhibitor JQ1 reversed this change and improved fibrosis. Analysis of dynamic gene expression revealed that *Gpx3* was significantly upregulated during cell differentiation into protective cells. *Gpx3* expression was affected by JQ1 treatment. Furthermore, *Gpx3* expression levels were negatively correlated with the different levels of fibrosis observed in the various treatment groups. Finally, we found that transcription factors *Jun*, *Fos*, *Atf3*, and *Egr1* were upregulated in protective cells, especially *Egr1* was predicted to be involved in the regulation of genes related to antioxidant stress and angiogenesis, suggesting a role in promoting differentiation into this cell phenotype.

**Conclusions:**

The scRNA-seq analysis was used to characterize the dynamic changes associated with fibroblast differentiation and identified *Gpx3* as a factor that might be involved in the regulation of myocardial fibrosis under cardiac pressure overload. These findings will help to further understanding of the mechanism of fibrosis and provide potential intervention targets.

## 1. Introduction

The heart compensates for increased workload through structural remodeling, which can ultimately lead to heart failure [1]. Myocardial fibrosis, as a form of cardiac remodeling, is a common process in many pathophysiologies, such as hypertension [2], myocardial infarction [3], and valvular disease [4]. In response to injury, myocardial fibroblasts show abnormal activation and secrete excessive extracellular matrix, resulting in chamber wall stiffness and diastolic dysfunction [5]. Many studies have revealed potential mechanisms associated with myocardial fibrosis. Myocardial fibrosis is the result of crosstalk between various types of cells, such as macrophages, which can secret pro- and antifibrotic factors [6], regulatory T cells, which can participate in regulating fibrosis by inhibiting excessive inflammation [7], and endothelial cells, which can secrete factors that interfere with the process of fibrosis [8]. Moreover, inflammatory reactions [9], oxidative stress [10], energy metabolism [11], apoptosis [12], and other mechanisms all play a role in the regulation of fibrosis. However, despite recent scientific advances, the current antifibrotic treatment options are still limited [13]. Therefore, exploring the mechanism of myocardial fibrosis and developing new therapeutic interventions are critical.

In recent years, single cell RNA sequencing (scRNA-seq) has become a standard tool for medical research that has been used in many fields [14]. Single cell sequencing allows for analysis of cell heterogeneity, intercellular communication, and cell developmental trajectories, which are important in the study of tissue development and pathology [15]. Several therapeutic targets for myocardial fibrosis, such as *Ddah1*, *Meox1*, and *Ctrhc1*, have been identified by single cell sequencing analysis and have provided novel theoretical targets for the treatment of myocardial fibrosis [16–18].

The purpose of this study was to explore the heterogeneity and activation of myocardial fibroblasts in a stress overload mouse model using scRNA-seq analysis. Our aim was to clarify some of the mechanisms of fibrosis and explore potential targets for treating myocardial fibrosis.

## 2. Materials and Methods

### 2.1. Analysis and Integration of scRNA-seq Data

The datasets analyzed in this study were obtained from the GEO database (https://www.ncbi.nlm.nih.gov/geo/). First, we filtered the presumed doublet cells through DoubletFinder (v2.0.3) [19]. R package Seurat (v4.0.5) [20] was used for data integration, cell filtration, normalization, clustering, and UMAP or tSNE dimensional reduction. Harmony (v0.1.0) [21] was used to remove the batch effect between groups. Overall, we excluded cells with nFeatures < 200, nFeatures > 3,000, and percent.mt > 10% for initial quality control. After clustering, the cell clusters with the most significant differences in nFeatures and percent.mt were further excluded.

### 2.2. Pseudotime Trajectory Analysis

Monocle2 was used for pseudotime analysis [22]. Cell progression genes were defined based on differential gene expression between Seurat clusters. “BEAM_res” function was used for analysis of gene dynamics during cell differentiation.

### 2.3. Gene Ontology (GO) Enrichment Analysis

Enriched GO terms in differentially expressed genes were identified using the clusterprofileR package (v0.5.0) [23] with the default parameters.

### 2.4. Hub Gene Analysis

First, differential genes were introduced into the STRING v11 database (https://string-db.org/) to obtain the PPI network. Then, hub genes were analyzed via Cytoscape v3.9.1 [24]. Notably, the hub genes were ranked by “degree.”

### 2.5. Gene Set Enrichment Analysis (GSEA)

The GSEABase (v1.56.0) package (http://www.bioconductor.org/packages/release/bioc/html/GSEABase.html) was used to complete GO and KEGG enrichment analysis of marker genes in all groups.

### 2.6. Transcription Factor Activity Evaluation and Transcription Factor Target Overrepresentation Analysis

The transcription factor activity of different cell states was predicted using the DoRothEA (v1.6.0) package [25] with default parameters. The transcription factors of gene sets related to angiogenesis and antioxidant stress were predicted by ChEA3 [26], and the gene sets were submitted online (https://maayanlab.cloud/chea3/). By comparing multiple libraries and scoring, the top 15 transcription factors were finally obtained by sorting with “mean rank.”

### 2.7. Receptor Ligand Interaction Analysis

We evaluated intercellular receptor ligand signals by NicheNet [27], fibroblasts were used as sender and endothelial cells as receiver, and the other parameters were default.

## 3. Results and Discussion

### 3.1. The scRNA-seq Analysis of Cardiac Fibroblasts (CFs) in a Transverse Aortic Coarctation (TAC) Model

**Figure 1:**
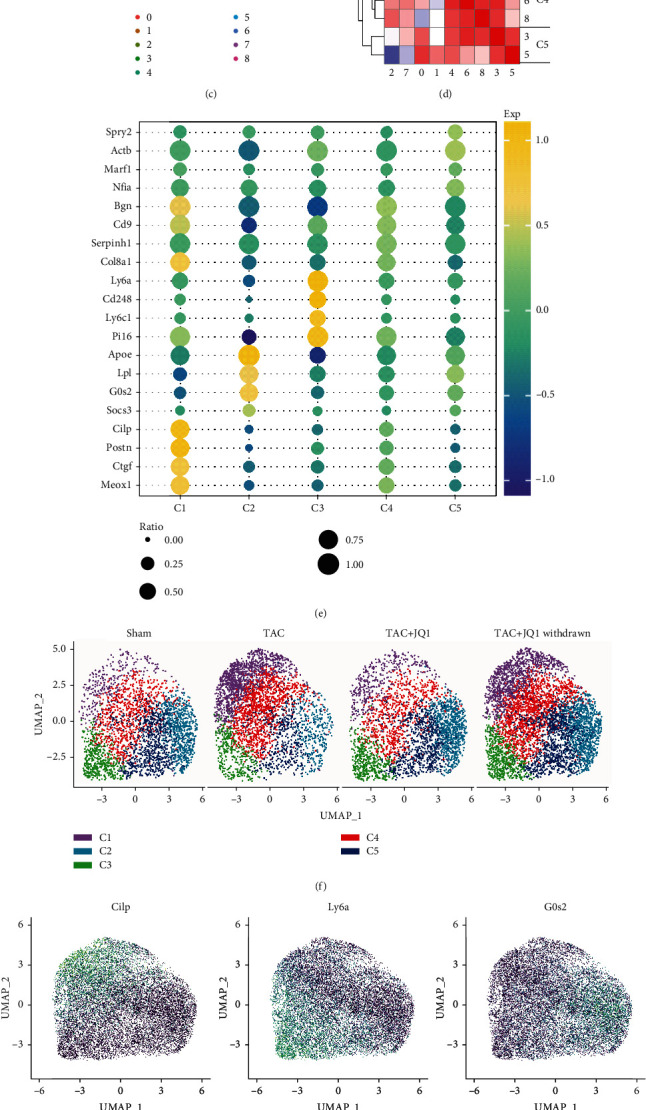
Overview of the fibroblast populations profiled in healthy and nonhealthy hearts. (a) Grouping and interventions in the single cell datasets. (b) The tSNE plot of nonmyocardial cells in 4 groups. (c) UMAP plot of CFs in four groups. A total of nine cell clusters were obtained. (d) Correlation heat map between clusters. The cell clusters were renamed C1 − C5 based on their correlation. (e) Relative expression levels of the marker genes for C1 − C5 shown by a bubble plot. (f) Unsupervised clustering of CFs visualized by groups. (g) FeaturePlot of some representative marker genes. (h) The proportion of C1 − C5 in each group.

Alexanian et al. [17] established TAC and Sham models in mice and administered JQ1 [28], a small molecule BET bromodomain inhibitor, 18 days later. Mice were treated with JQ1 for one month, then JQ1 treatment was stopped in one cohort of mice for 14 days. Finally, the heart samples (*n* = 2) of four groups were harvested for single cell sequencing (Figure 1(a)). First, we carried out quality control on the data and identified a total of 58,175 cells; after quality control, the data had no significant batch effect, and the nFeatures and percen.mt of the data were within a reasonable range (Supplementary Figure 1(a, b)). After dimensionality reduction and clustering, these cells were annotated according to marker genes (Supplementary Table 1). A total of 14 cell subtypes were obtained (Figure 1(b)), in which activated CFs expressed high levels of profibrotic factors such as *Postn*, *Sparc*, and *Cilp* [29–31]. Ly6a^+^ CFs expressed high levels of the stem cell marker *Ly6a* [32] and fibrotic inhibitor *Pi16* [33]. Another group of CFs (G0s2^+^ CFs) showed high expression of *G0s2*, which blocks lipolysis and inhibits the cell cycle [34], as well as expressing the Antioxidant stress-related gene *Gpx3* [35] and cardioprotective gene *Adm* [36] (Supplementary Figure 1(c)).

To further explore CF heterogeneity in cardiac fibrosis, we defined additional CF populations using unsupervised graph-based clustering and UMAP. In total, nine cell clusters were identified in the whole dataset (Figure 1(c)). We extracted the top marker genes with the largest standard deviation of each cell cluster to calculate the correlation. Based on this, we reclassified these nine cell clusters into five CF subgroups, C1-C5 (Figure 1(d)). C1 and C4 displayed high expression levels of profibrotic genes such as *Postn*, *Ctgf*, and *Meox1*, indicating that these cell groups had a stronger fibrotic phenotype. C2 overexpressed *Apoe*, *G0s2*, and *Lpl*, which are thought to be related to fat metabolism, while C3 overexpressed *Ly6a*, *Pi16*, and *Cd248*. Gene expression levels in the C4 and C5 subgroups were not as high as those observed in the C1-C3 subgroups and were therefore considered to be cell subsets in a transition state (Figures 1(e) and 1(g)). Although we did not observe significant batch effects, there were significant differences in cell distribution among the different groups (Figure 1(f)). As shown in Figure 1(h), the number of cells in C1 and C4 was significantly increased in the TAC group, decreased after treatment with JQ1, and partially attenuated by JQ1 withdrawal. Since changes in the number of cells in the C2, C3, and C5 subgroups were the opposite of those found in C1 and C4, we concluded that these two types of cells played different roles in CFs under pressure overload.

### 3.2. JQ1 Treatment Attenuates Oxidative Stress and Fibrosis in CFs

**Figure 2:**
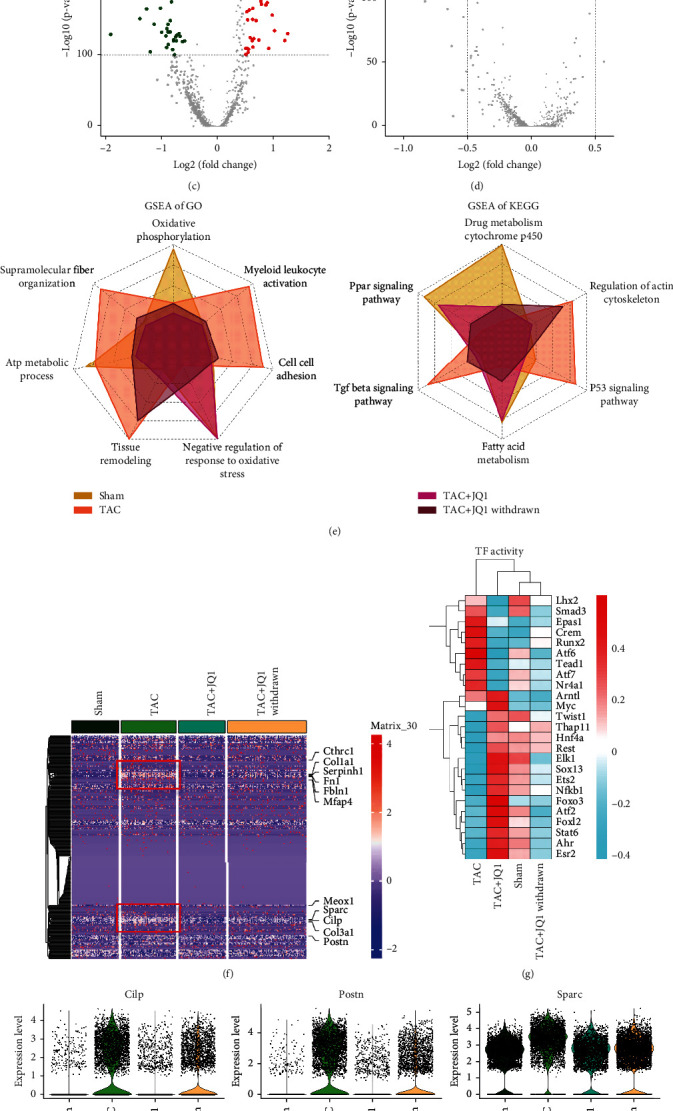
Characteristics of CFs in each treatment group. (a)–(d) Volcano plot showing the marker genes of each group. Marker genes with *p* value < 1*e*^100^ and |log2fc| > 0.5 are labelled. (e) GSEA enrichment analysis of GO and KEGG terms in each group is displayed by radar plot. (f) Heat map of genes related to fibrosis. (g) Heat map showing the relative levels of transcription factor activity in each group. (h) Volcano plot showing the expression of representative profibrotic genes in each experimental group.

Since JQ1 can improve fibrosis and enhance cardiac function, examining the characteristics of the four treatment groups may further our understanding of the mechanisms of fibrosis. We analyzed the marker genes of each group and displayed the data by volcano plot. The TAC group showed high expression of profibrotic-related genes such as *Ctgf*, *Cilp*, and *Thbs1*, as well as high expression of *Actn1* and *Csrp2*, which are associated with actin contraction (Figure 2(b)). After JQ1 treatment, the fibrotic-related genes were significantly downregulated, while upregulation of the heart protective genes *Eef2* [37] and *Dusp1* [38] was observed (Figure 2(c)). To some extent, JQ1 treatment restored the gene expression pattern in the TAC group to that observed in the Sham group (Figure 2(a)). Withdrawal of JQ1 resulted in downregulation of the protective and fibrogenic genes, which might explain lower levels of heart protection in the JQ1 withdrawal group compared to the JQ1 treatment group (Figure 2(d)). Next, we obtained the marker genes of each group with the parameters of “min.pct =0.01, logfc.threshold = 0” and sorted them by “log2FC.” Then, we performed GO and KEGG enrichment analysis of the marker genes for each group and displayed them with radar plot (Supplementary Table 2). GO terms related to fibrosis, such as “Supramolecular Fiber Organization” and “Cell Cell Adhesion,” were significantly upregulated in the TAC group, but improved after JQ1 treatment. In addition, we found that JQ1 significantly improved oxidative stress, and no significant differences in the enrichment level of the GO term “Negative Regulation Of Response To Oxidative Stress” were observed between the TAC + JQ1 and Sham groups (Figure 2(e)). Similar results were found by KEGG enrichment analysis. For example, the gene set “Drug Metabolism Cytochrome P450” related to antioxidative stress and including genes such as *Gstm1*, *Gsta3*, and *Gpx3* was found to be upregulated in the JQ1 treatment group compared to the TAC group.

Next, we studied the effect of JQ1 therapy on fibrosis. We obtained a gene list of fibrosis-related GO terms (Supplementary Table 3) and used heat map to compare their expression in the four groups. We found that fibrosis-related genes in the TAC group were significantly upregulated compared to those in the Sham group, and that JQ1 treatment reversed this outcome, while withdrawal of JQ1 weakened the JQ1-induced improvement in fibrosis (Figure 2(f)). The expression of representative fibrogenic genes is shown in Figure 2(h) and is consistent with the heat map data. Because transcription factors regulate gene expression, they play an important role in the development of diseases. Thus, we analyzed the activity of transcription factors in each group. Our data revealed significant upregulation of *Atf6*, *Runx2*, *Crem*, and *Tead1* in the TAC group, with *Atf6* and *Runx2* associated with oxidative stress [39, 40], while *Crem* and *Tead1* were involved in promoting fibrosis [41, 42]. Transcription factor activity levels in the TAC + JQ1 and Sham groups were very similar, with *Atf2*, *Elk1*, *Ets1*, and *Esr1* associated with inhibition of oxidative stress [43–45]. In the TAC + JQ1 withdrawal group, both protective and harmful transcription factor activities were impaired (Figure 2(g)).

### 3.3. Differential Fates of CF Differentiation

**Figure 3:**
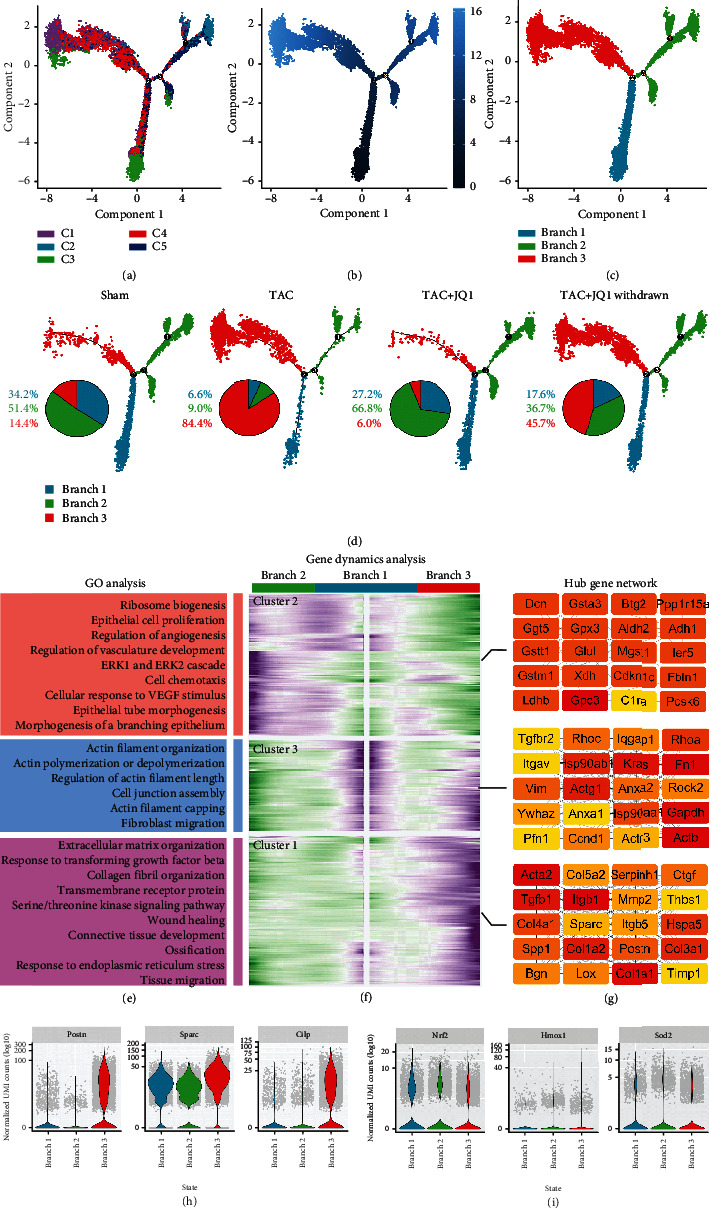
Simulation of the developmental trajectory of CFs and analysis of gene expression patterns inferred by Monocle2. (a) Pseudotime trajectory analysis of fibroblasts, using significantly regulated genes between C1 and C5. (b) Time series inference of cell trajectories. The color gradient indicates pseudotime progression. (c) Cell trajectories were renamed according to three main branches. (d) Cell trajectories of each group. Pie chart showing the proportion of cells in each branch of each group. (e) GO biological process terms enriched in each gene cluster. (f) Heat map hierarchical clustering showing differentially expressed genes together with the pseudotime curve. Color key from green to purple indicates relative expression levels from low to high. (g) Gene network displaying the top 20 hub genes from each cluster. (h, i) Expression of representative profibrotic genes and antioxidative stress genes in each branch.

Our data indicated that the four CF treatment groups had different fibrotic phenotypes and biological functions. Next, we examined the differentiation trajectories of CFs and compared the differences between the groups through pseudotime analysis. In the cell trajectory, we identified three main branches, which we named branches 1 − 3 (Figure 3(c)). As a putative starting point of development, branch 1 was mainly composed of C4, which highly expressed the stem cell marker, *Ly6a*. Two distinct differentiation fates were derived from branch 2. Branch 2 was mainly composed of C2 and C5, while branch 3 was mainly composed of C1 and C4, which exhibited high expression levels of fibrogenic genes (Figures 3(a) and 3(b)). We evaluated the proportion of branches in each group and found that the TAC group was dominated by branch 3, suggesting that branch 3 was closely related to the fibrotic phenotype. In contrast, the JQ1 treatment group showed a significant reduction in the proportion of branch 3, even larger than that observed in the Sham group (Figure 3(c)). A higher proportion of branch 2 was observed in the JQ1 treatment group relative to the Sham group, suggesting that JQ1 treatment altered the fate of CFs.

To explore the mechanism of CF differentiation, we evaluated the gene dynamics of two different cell fates. Genes were divided into three clusters according to their expression patterns: genes of cluster 2 were gradually upregulated in branch 2, while genes of cluster 1 and cluster 3 were gradually upregulated in branch 3 (Figure 3(e)). Genes of cluster 2 were found to be mainly enriched in angiogenesis and ribosome composition (Supplementary Table 4). We further analyzed its PPI network using Cytoscape and sorting genes by “Eccentricity.” Of the selected top 20 genes, *Gstt1*, *Gpx3*, *Gstm1*, *Gpc3*, and *Ggt5* were widely considered to be related to antioxidative stress. The gene function of cluster 3 was mainly enriched in actin filament organization, and its top 20 genes (sorted by degree) included *Actb*, *Actg1*, and A*ctr3*. The top 20 genes (sorted by degree) of cluster 1 included *Acta2*, *Tgfb1*, *Postn*, *Ctgf*, and *Col1a1*, which are well-established fibrogenic genes. The GO enrichment analysis data were consistent with these findings. The expression of genes related to fibrosis and oxidative stress in each branch was evaluated by vlnplot. We found that branch 2 had reduced expression of fibrosis-related genes and increased expression of genes associated with antioxidative stress (Figures 3(f) and 3(g)).

### 3.4. Gpx3 May Be Involved in Regulating the Differentiation of CFs

**Figure 4:**
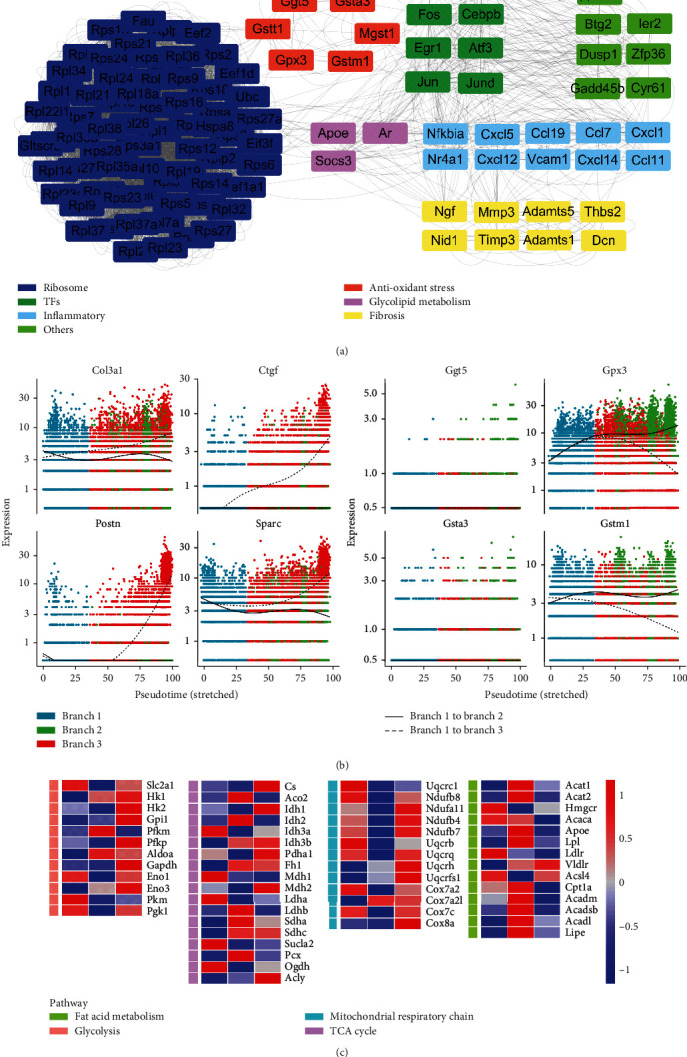
Examination of the function of upregulated genes in branch 2. (a) The top 100 hub gene network in gene cluster1 (described in Figure 2(f)), showing genes that were upregulated in branch 2. The function of these genes was classified and color-coded based on reports in the literature. (b) Differences in expression of *Gpx3*, *Gstm1*, *Gsta3*, *Ggt5*, and fibroblast-promoting genes in the different cell trajectories. (c) Heat map showing the relative expression levels of key genes related to fatty acid (FA) signals, tricarboxylic acid (TCA) cycle, glycolytic pathway, and mitochondrial respiration in each branch.

According to the results described above, genes of cluster2 that were upregulated in branch 2 were associated with angiogenesis and antioxidative stress. In order to further explore its specific mechanism, we selected the top 100 genes in the PPI network of cluster 2 and classified each gene according to reports in the literature (Figure 4(a)). Genes labelled blue are related to ribosomal synthesis, while yellow-labelled genes may be involved in the negative regulation of fibrosis. The dark-green-labelled genes encode transcription factors that play a protective role in a variety of diseases. In addition, gene sets labelled red such as *Gpx3*, *Ggt5*, and *Gstm1* were considered to be associated with antioxidative stress, and the differential expression of these genes in the cell trajectories was evaluated further. The expression of *Gpx3*, *Gstm1*, *Ggt5*, and *Gsta3* showed different trends in the two cell fates, in contrast to *Postn*, *Sparc*, and *Ctgf* expressions (Figure 4(b)). Compared with other red-labelled genes, the expression of Gpx3 in the cell track changed more significantly (Supplementary Figure 2(a)) and compared its expression pattern with *Postn* and *Col1a1*. *Gpx3* was mainly expressed in branch 2, while *Postn* and *Col1a1* were predominantly expressed in branch 3. Furthermore, *Gpx3* was downregulated in the TAC group and upregulated after treatment with JQ1, whereas withdrawal of JQ1 weakened the JQ1-induced upregulation of *Gpx3*. Meanwhile, the expression trend of the profibrotic gene *Postn* and *Col1a1* was the opposite of *Gpx3* (Supplementary Figure 2(b)). These data suggested that *Gpx3* may inhibit CFs from differentiating into branch 3 and may promote differentiation into branch 2. In addition, we found that *Apoe*, *Lpl*, and *Socs3* were also highly expressed in branch 2, suggesting that there might be metabolic differences between the branches. Thus, we evaluated the expression of metabolism-related genes in each group and found that the fatty acid metabolism levels of branch 2 were significantly increased, while the glycolysis and mitochondrial respiration levels were decreased, especially mitochondrial respiration. Of note, branch 3 had the highest glycolysis level among all branches (Figure 4(c)). We also evaluated the levels of pentose phosphate pathway, glycogen synthesis, glycogen decomposition, and gluconeogenesis, and the results showed no significant difference (Supplementary Figure 3(a)).

### 3.5. CFs of Branch 2 Are Involved in Promoting Angiogenesis

**Figure 5:**
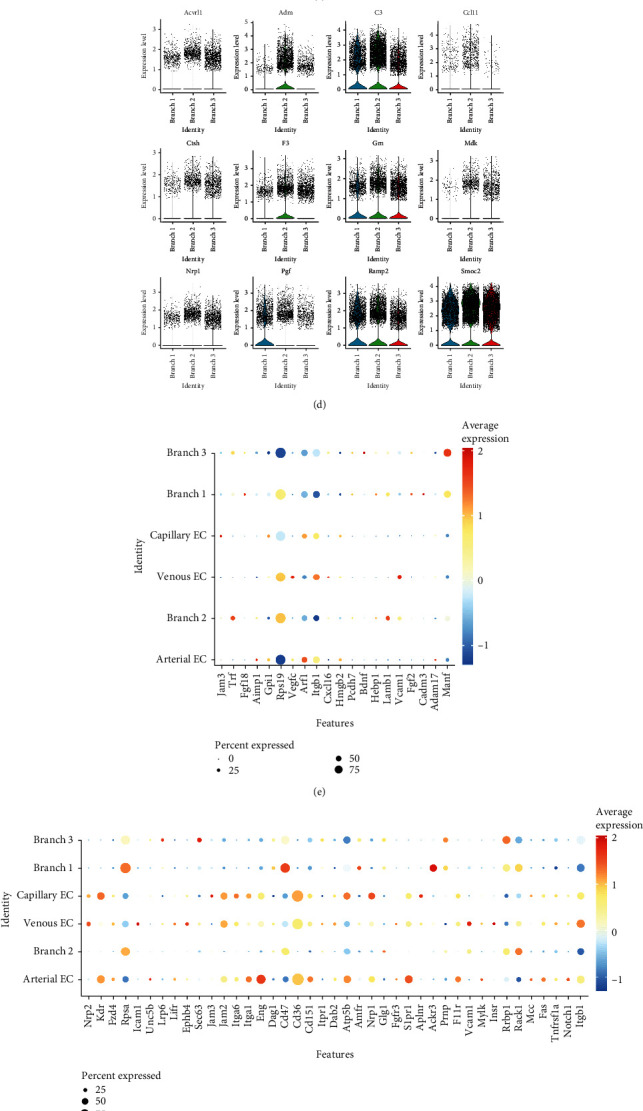
CFs participate in angiogenesis. (a)–(c) The secretory protein gene set and the angiogenic gene set were, respectively, intersected with the marker genes of each branch. Four overlapping genes were found in branch 1, 12 in branch 2, and five in branch 3. (d) Vlnplot showing the expression of 12 genes encoding angiogenesis-promoting secretory proteins in branch 2. (e) Top ligands in each cell type obtained by NicheNet analysis. (f) Top receptors in each cell type. (g) L-R pairs between cell types.

It is interesting to note that, as shown in Figure 3(e), genes that were gradually upregulated in branch 2 were mainly associated with angiogenesis. Because CFs are unable to promote the sprouting of endothelial cells directly, we assumed that CFs promote angiogenesis by secreting proteins. Thus, we downloaded 3970 genes encoding secretory proteins (Supplementary Table 5), as well as angiogenesis-promoting genes (Supplementary Table 6), and intersected them with the marker genes of each branch. We found that four marker genes in branch 1 encoded angiogenic secretory proteins, compared to five in branch 3, and 12 in branch 2 (Figures 5(a)–5(c)).  Figure 5(d) shows the expression of these 12 genes through vlnplot. Our data indicated that branch 2 had the largest number of genes encoding proteins that promote angiogenesis compared with the other two branches, which was consistent with the results described above. Next, we selected CFs and endothelial cell subsets and mapped the branch labels of the trajectory of CFs into these cells (Supplementary Figure 3(b)). Then, the NicheNet package was used to explore the ligand-receptor (L-R) signal communication between the three branches of CFs and endothelial cells. *Trf* and *Lamb1* were found to be highly expressed in branch 2, while *Manf* and *Bdnf* were highly expressed in branch 3. Based on the L-R pairs shown in Figure 5(g), we identified the corresponding receptors. *Itga6* was identified as the receptor of *Lamb1* and was found to be highly expressed in capillary endothelial cells, while *Fzd4* was the receptor of *Trf*, which was shown to be enriched in arterial endothelial cells. Both receptors have been shown to be associated with angiogenesis. *F11r*, the receptor of *Bdnf*, was expressed in arterial endothelial cells and has been reported to be associated with atherosclerosis. *Sec63* was found to be the receptor of *Manf* and was widely expressed in CFs of branch 3.

### 3.6. Egr1 May Be Involved in Reprogramming the Fate of CFs

**Figure 6:**
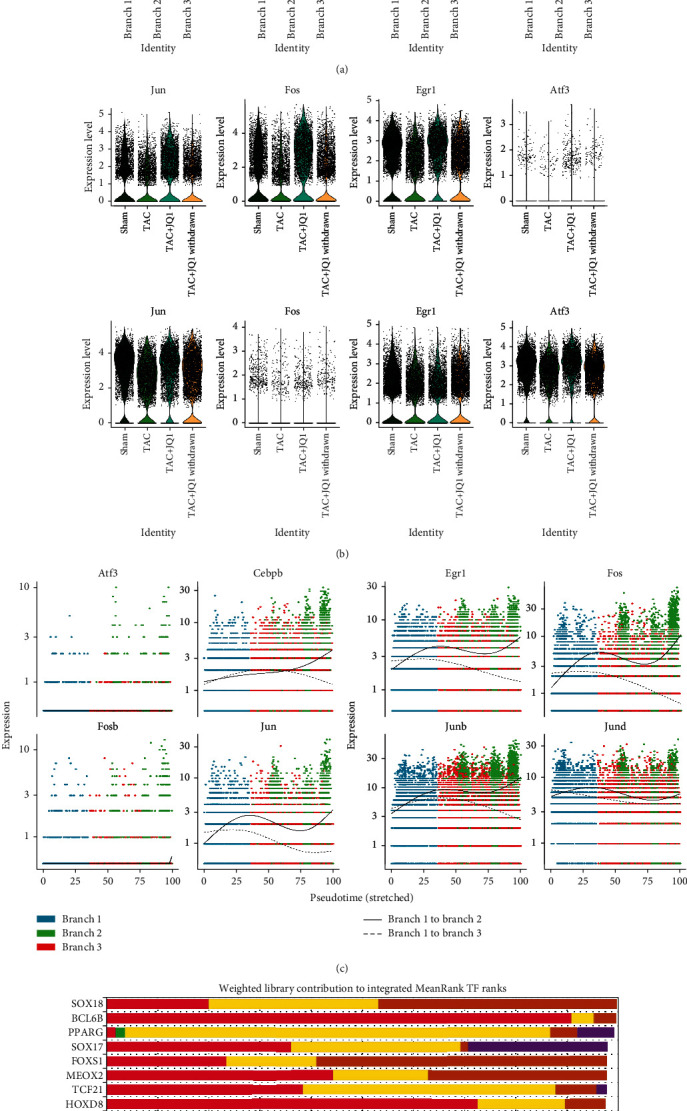
Comprehensive characterization of *Clec3b* expression in fibroblasts. (a) Expression of the transcription factors *Jun*, *Fos*, *Fosb*, *Junb*, *Jund*, *Cebpd*, *Atf3*, and *Egr1* in each branch are displayed by vlnplot. (b) Expression of these eight transcription factors in each treatment group. (c) Expression of these 8 transcription factors in each cell trajectory. (d) Top 15 transcription factors predicted by ChEA3, sorted by average integrated ranks across the libraries.

In the gene network of branch 2, multiple genes encoding transcription factors were upregulated and served as the core of the network (Figure 4(a)). Transcription factors play an important role in the pathophysiological process of disease, and thus, in-depth examination of these transcription factors will improve our understanding of fibroblast differentiation. With the exception of *Atf3* and *Fosb*, these transcription factors were widely expressed in CFs and were significantly upregulated in branch 2 (Figure 6(a)). The expression levels of these transcription factors were found to be decreased in the TAC group, but increased after JQ1 treatment, and decreased after JQ1 withdrawal. These findings were consistent with the *Gpx3* data, but in contrast to the *Postn* data (Figure 6(b)). Similarly, the changing trends of these transcription factors on cell trajectories were consistent with *Gpx3* but opposite to *Postn* (Figure 6(c)). Since the expression levels of these transcription factors were consistent with those of *Gpx3*, *Gstm1*, and genes encoding angiogenesis-promoting secretory proteins, we further predicted the transcription factors of these genes by ChEA3. *Egr1* was identified from the top 15 predicted transcription factors and was also found to be highly expressed in branch 2. Based on the above results, we speculated that *Egr1* may be an upstream transcription factor that has a role in improving oxidative stress and promoting angiogenesis.

## 4. Discussion

This study fully characterized the heterogeneity of CFs in a mouse TAC model, as well as the cell development trajectory through scRNA-seq analysis. Based on scRNA-seq analysis, we propose for the first time that there are two distinct cell differentiation fates for CFs under pressure overload. Fibroblasts expressing the stem cell marker *Ly6a* served as the starting point of differentiation (branch 1), from which two types of cell differentiation trajectories were derived, one of which was involved in the promotion of fibrosis (branch 3) and the other, which was involved in antioxidative stress and angiogenesis (branch 2). In the nonstress state, CFs are mainly differentiated into branch 2. However, under pressure overload conditions, CFs increased their differentiation into branch 3, which promoted fibrosis. Treatment with JQ1 significantly restored the fate of branch 2 and significantly alleviated fibrosis levels, which proved to be the powerful therapeutic effect of JQ1.

GSEA analysis revealed that the PPAR signaling pathway in the TAC heart was significantly downregulated compared with the Sham group. PPARs belong to the nuclear hormone receptor superfamily [46, 47]. PPAR*γ* expression is closely associated with myocardial fibrosis [48, 49], due to its involvement in regulating inflammation, energy metabolism, and oxidative stress [50–53]. PPAR agonists are expected to be beneficial in antimyocardial fibrosis therapy [54]. Furthermore, CFs in branch 2 were found to express high levels of *Apoe*, *Lpl*, and *Socs3*, which are related to lipid metabolism. To evaluate metabolic differences in fibroblast differentiation, we evaluated the expression of key genes of fatty acid metabolism, glycolysis, the TCA cycle, and mitochondrial respiratory chain in cell development trajectories. We found that during the differentiation of fibroblasts into nonprotective branch 3, glycolysis levels gradually increased and were accompanied by a reduction in fatty acid metabolism. Thus, the balance of normal cardiac energy substrates was dysregulated, and so, under pressure overload, fibroblasts tend to be fueled by the glycolytic pathway. Many studies have reported that excessive glycolysis is involved in the process of organ fibrosis. Thus, inhibition of glycolysis can inhibit the activation of fibroblasts and improve organ fibrosis [55–58]. This change of energy metabolism may be an adaptive measure under hypoxia, as cells tend to undergo glycolysis in the absence of oxygen [59, 60]. Thus, we speculate that restoring normal levels of fatty acid metabolism may be a potential treatment for fibrosis.

Next, we assessed the level of oxidative stress in fibroblasts in each group. We found that the ability to resist oxidative stress was significantly impaired in the TAC group. Oxidative stress also plays an important role in the differentiation of fibroblasts. CFs that differentiated into branch 2 exhibited higher antioxidative stress abilities. However, differentiation into branch 3 was accompanied by a reduction in antioxidative stress ability. JQ1 treatment promoted the differentiation of cells into branch 2 and significantly restored the expression levels of antioxidative stress-related genes in fibroblasts, especially *Gpx3*. However, the withdrawal of JQ1 partially weakened this protective effect. These results indicated that oxidative stress was involved in the regulation of fibrosis, while JQ1 was found to have an anti-fibrotic role, at least in part through the regulation of oxidative stress [61–64]. Notably, *Sdha* and *Sdhc*, key genes involved in mediating succinic acid metabolism, were significantly reduced in fibroblasts from the TAC heart. However, previous studies have reported that *Sdh*-mediated succinic acid oxidation drives mitochondria to produce ROS, which is involved in the promotion of oxidative stress injury [65, 66]. This may be the compensation mechanism of energy metabolism reprogramming to cope with oxidative stress [67–70].

In addition, we observed an interesting phenotype. We found that, compared with other branches, branch 2 had a higher ability to promote angiogenesis, due to increased production of secreted proteins that promote angiogenesis, such as *C3*, *Mdk*, *Pgf*, and *Ramp2* [71–73]. We analyzed the L-R signals between CFs of the three branches and endothelial cells via NicheNet and obtained similar results. We identified *Trf-Fzd4* and *Lamb1-Itga6* as the L-R pairs between branch 2 and ECs. Studies have shown that *Fzd4*, as a receptor of *Wnt*, participates in promoting angiogenesis [73], while *Itga6* promotes endothelial morphogenesis through regulation of *Cxcr4* [74]. *Bdnf-F11r* was identified as a L-R pair between branch 3 and endothelial cells. A role for *F11r* in promoting atherosclerosis has previously been reported [75]. Our results indicated that branch 2, as one of the potential fates of fibroblast differentiation, has an increased ability to promote angiogenesis. The concept of fibroblasts having a role in the promotion of angiogenesis is not new. Fibroblasts reportedly exhibit characteristics associated with promoting angiogenesis on the third day after myocardial infarction but have an antiangiogenesis role on day 7 [76]. This mechanism also plays an important role in tumor angiogenesis [77]. Our results not only support this concept but also clearly show that CFs that promote angiogenesis constitute one of the branches of CF fate. At the same time, this branch has a strong antioxidative stress capability, as well as increased fatty acid metabolism.

Finally, we evaluated the expression of transcription factors such as *Jund*, *Fos*, *Atf3*, and *Egr1* in branch 2. Expression of these transcription factors was reduced in the TAC group, but improved following JQ1 treatment. Studies have shown that these transcription factors play a beneficial role in many pathophysiological processes. Overexpression of *Atf*, for example, can alleviate ischemia-reperfusion injury in the heart by reducing oxidative stress [78]. Indeed, *Atf3* may be a target of *Nrf2* and may therefore be involved in the regulation of oxidative stress [78]. *JunD*, a member of the AP-1 transcription factor family, plays a protective role in diabetic cardiomyopathy through the regulation of antioxidative stress [79]. Similarly, *Cebpd* has been shown to be involved in inhibiting oxidative stress and maintaining mitochondrial function [80, 81]. Here, we predicted the transcription factors of angiogenesis-promoting genes including *Gpx3*, *Gstm1*, and *Gstt1*. Among the predicted transcription factors, *Egr1*, which was also enriched in branch 2, was of particular interest. Multiple studies have reported that *Egr1* promotes collagen synthesis and fibrosis [82]. *Egr1* has also been shown to lead to upregulation of oxidative stress levels and has been associated with a variety of adverse outcomes, such as myocardial fibrosis, renal fibrosis, pulmonary hypertension, and arrhythmia after myocardial infarction [83–86]. However, in a model of myocardial ischemia-reperfusion injury, remote ischemic preconditioning was found to improve myocardial ischemia-reperfusion injury, through the protective role of *Egr1* against apoptosis via the Jak-Stat pathway [87]. In most studies, *Egr1* has been examined in the context of an acute pathological model. However, in the cardiac stress overload model, *Egr1* was been studied in the context of myocardial hypertrophy. To date, there are no reports describing the expression and function of *Egr1* in CFs in a chronic TAC model. However, Cupesi et al. reported that in a TAC model, *Egr1* was upregulated after week 1, but downregulated after week 3 [88]. Based on our scRNA-seq analysis of the TAC model, we found that *Egr1* expression levels were downregulated in fibroblasts in the TAC model. However, JQ1 treatment led to upregulation of *Egr1* expression levels, which was accompanied by improved fibrosis. Thus, we propose that *Egr1* expressed by fibroblasts may play a protective role in the model of chronic cardiac pressure overload. Our results show that the function of Egr1 may be inconsistent with most literature reports, which is worthy of further exploration. Finally, this study was carried out based on the analysis of single-cell sequencing data and has not been verified by experiments, which is the deficiency of this study.

## 5. Conclusions

This study found that there were two distinct cell fates in CFs. One fate may be related to angiogenesis and antioxidative stress, accompanied by metabolic reprogramming. Furthermore, *Gpx3* and *Egr1* may be involved in promoting the differentiation of CFs into a protective state.

## Data Availability

The dataset of the transverse aortic coarctation (TAC) model in mice was downloaded from the GSE155882 dataset.

## Additional Points

*Code Availability*. Codes for R analysis of data presented in this study are available on GitHub (https://github.com/Leeguoxi/scRNA_TAC).

## Supplementary Materials

Supplementary Materials Supplementary Figure 1: (a) the vlnplot shows the nFeatures and percent.mt of all cell subsets. (b) Unsupervised clustering of all cells visualized by groups, which showed that there was no obvious batch effect. (c) FeaturePlot showed the markers of fibroblast. Supplementary Figure 2: (a) the expression of *Gpx3*, *Gstm1*, *Ggt5*, *Gsta3*, *Mgst1*, and *Gstt1* in three branches of the trajectory. (b) Comparative analysis of expression patterns of *Gpx3*, *Col1a1*, and *Postn*. Supplementary Figure 3: (a) heat map of genes related to glycogen synthesis, glycogen decomposition, and pentose phosphate pathway. (b) We renamed the fibroblasts with branch labels and then selected subgroups for reclustering before performing receptor ligand interaction analysis. Supplementary Table 1: top 20 markers of all subsets. Supplementary Table 2: GSEA results of KEGG and GO. Supplementary Table 3: list of fibrogenic genes. Supplementary Table 4: GO analysis of three branches. Supplementary Table 5: list of gene encoding secreted protein. Supplementary Table 6: list of angiogenesis genes.
