# Supplementary material for: Gpx3 and Egr1 Are Involved in Regulating the Differentiation Fate of Cardiac Fibroblasts under Pressure Overload
Source: Oxid Med Cell Longev. 2022 Jun 28;2022:3235250. doi: 10.1155/2022/3235250 (PMC9256463; doi:10.1155/2022/3235250)
Supplement: Supplementary Materials — Supplementary Figure 1: (a) the vlnplot shows the nFeatures and percent.mt of all cell subsets. (b) Unsupervised clustering of all cells visualized by groups, which showed that there was no obvious batch effect. (c) FeaturePlot showed the markers of fibroblast. Supplementary Figure 2: (a) the expression of Gpx3, Gstm1, Ggt5, Gsta3, Mgst1, and Gstt1 in three branches of the trajectory. (b) Comparative analysis of expression patterns of Gpx3, Col1a1, and Postn. Supplementary Figure 3: (a) heat map of genes related to glycogen synthesis, glycogen decomposition, and pentose phosphate pathway. (b) We renamed the fibroblasts with branch labels and then selected subgroups for reclustering before performing receptor ligand interaction analysis. Supplementary Table 1: top 20 markers of all subsets. Supplementary Table 2: GSEA results of KEGG and GO. Supplementary Table 3: list of fibrogenic genes. Supplementary Table 4: GO analysis of three branches. Supplementary Table 5: list of gene encoding secreted protein. Supplementary Table 6: list of angiogenesis genes. [file 3235250.f1.zip › Supplementary Figure (2).docx]

Supplementary Figure 1: (a) The vlnplot shows the nFeatures and percent.mt of all cell subsets. (b) Unsupervised clustering of all cells visualized by groups, whitch showed that there was no obvious batch effect. (c) Featureplot showed the markers of fibroblast.


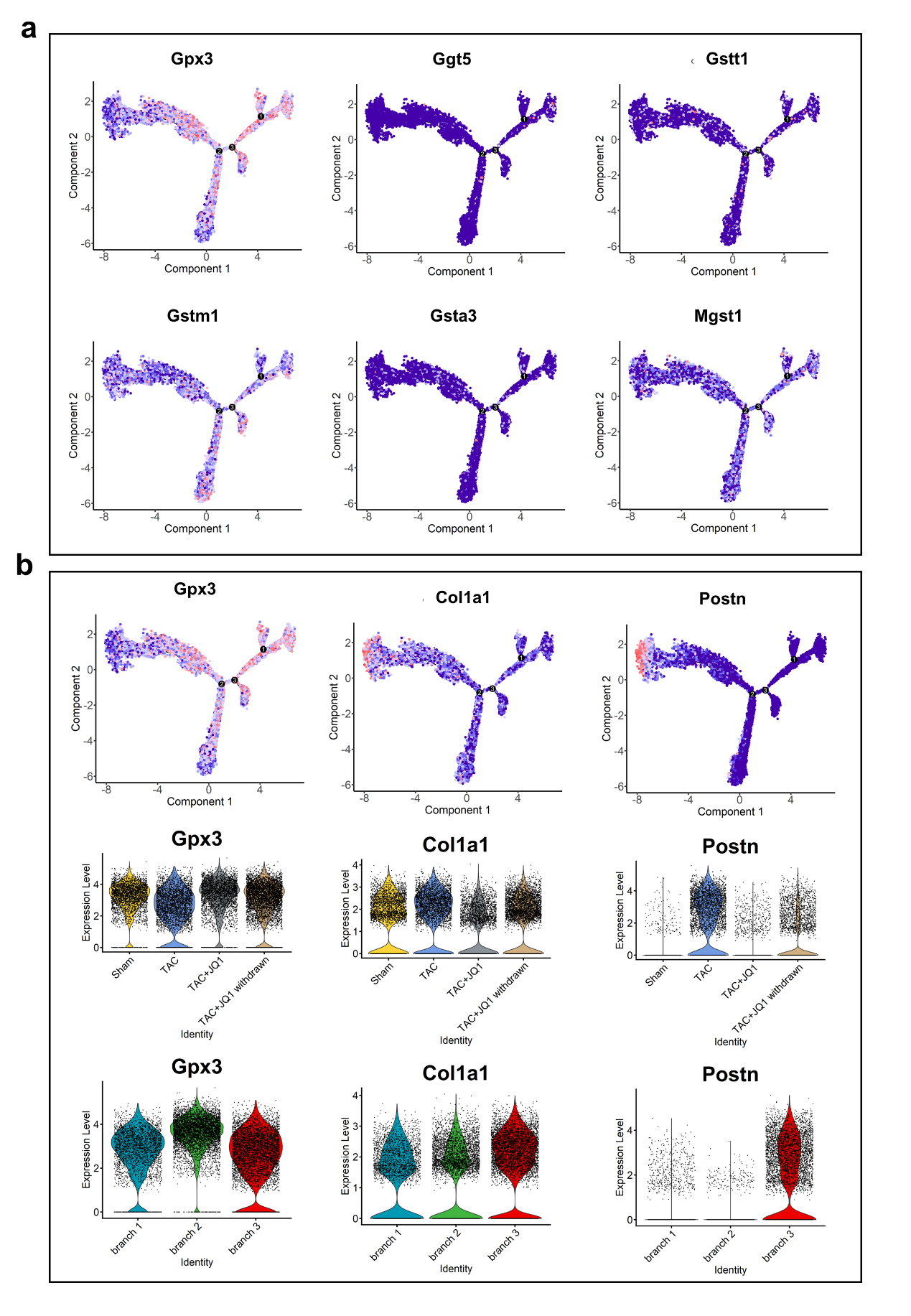
 Supplementary Figure 2: (a) The expression of *Gpx3*, *Gstm1,* *Ggt5, Gsta3, Mgst1, Gstt1* in three branches of the  trajectory. (b) Comparative analysis of expression patterns of *Gpx3*, *Col1a1* and *Postn*.


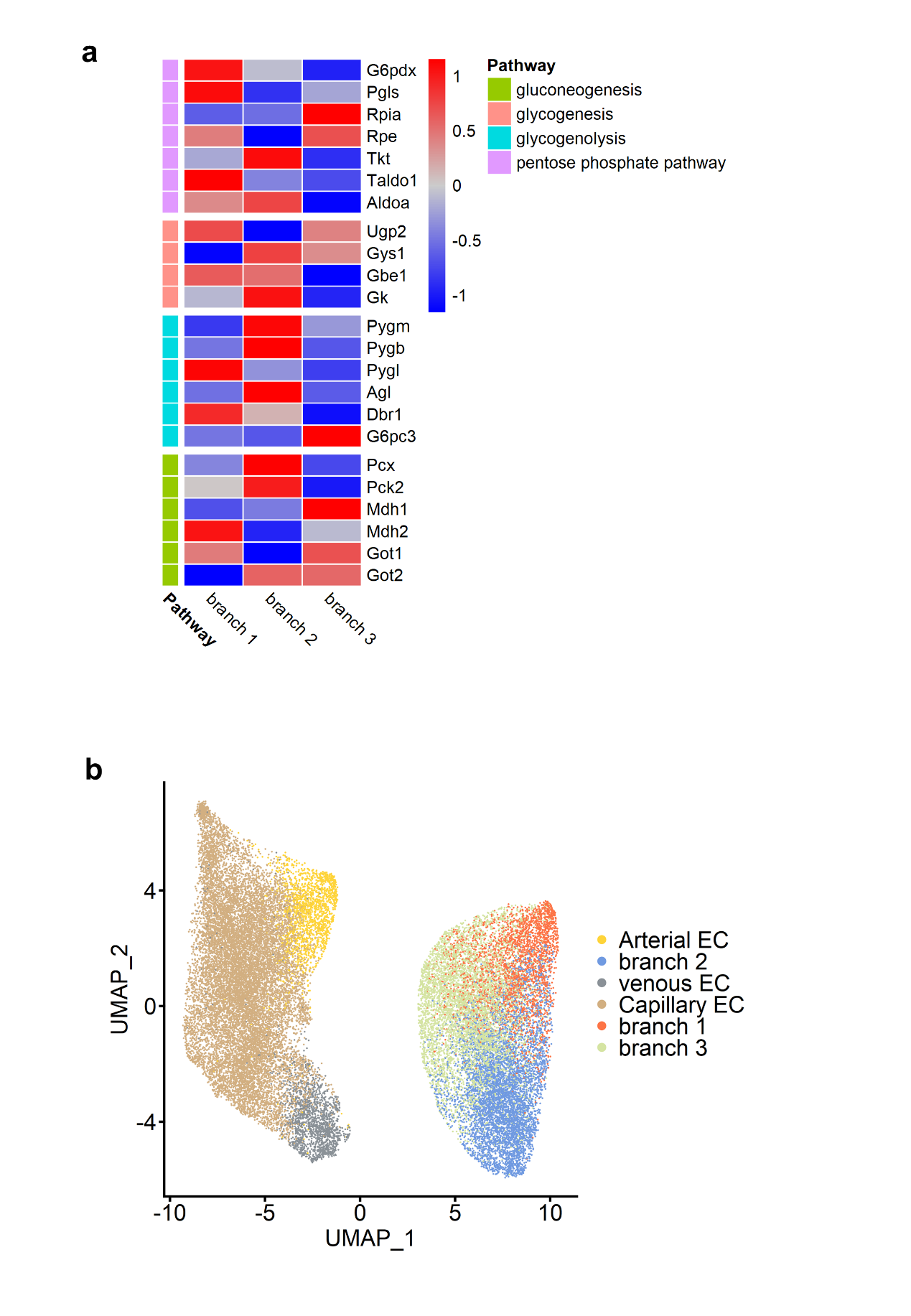
 Supplementary Figure 3: (a) Heat map of genes related to glycogen synthesis, glycogen decomposition and pentose phosphate pathway. (b) We renamed the fibroblasts with branch labels , and then selected subgroups for re-clustering before performing receptor ligand interaction analysis.
